# A nonlinear fractional-order damage model of stress relaxation of net-like red soil

**DOI:** 10.1038/s41598-021-02317-9

**Published:** 2021-11-25

**Authors:** Mingwu Wang, Xinyu Xu, Qiuyan Liu, Yingxun Ding, Fengqiang Shen

**Affiliations:** 1 School of Civil and Hydraulic Engineering, Hefei University of Technology, 193 Tunxi Road, Hefei, 230009 China; 2 School of Architectural Engineering, Tongling University, Tongling, 244000 China

**Keywords:** Natural hazards, Solid Earth sciences, Mathematics and computing

## Abstract

It is essential to precisely describe the nonlinear characteristics of the stress relaxation behavior to ensure the long-term stability of geotechnical structures in the net-like red soil. A novel damage model of variable fractional-order was discussed here to accurately analyze the progress of stress relaxation for the net-like red soil. Moreover, unsaturated triaxial experiments on stress relaxation under a step-loading mode were performed to identify model parameters and investigate the nonlinear relaxation characteristics of the net-like red soil. The feasibility and validity of the proposed model were furthermore verified by comparisons with the experimental results and fitting curves obtained from the Nishihara model and the generalized Kelvin model. Results show that the analytical result by the proposed model is consistent with the measured data, and the proposed model can better depict the nonlinear characteristics of stress relaxation relative to other analytical models. It can better exhibit the relaxation evolution of soil compared with the conventional models.

## Introduction

The net-like red soil, also known as lateritic red earth, reticulate red clay, or vermiculated red soil^1,2^, is found widely in southern China. It comprises the white vein and red matrix and is often used as the bearing layer of the foundation of geotechnical structures. However, previous researches on the net-like red soil were mainly focused on particle size^3^, clay mineral composition^4^, and climate significance^5^, rarely on rheological properties of mechanical behaviors, especially of stress relaxation. As we know, relaxation is a primary time-dependent behavior of clay, which plays a significant role in the long-term stability of geotechnical structures and soil-structure interaction^6,7^. However, previous studies on the stress relaxation of soil were much less than on the creep behavior because a constant strain in the stress relaxation experiment is relatively difficult to hold. It needs a long testing period and high-precision equipment^8^. Consequently, a deep insight into stress relaxation behavior by numerical analysis can bring about a thorough comprehension of the rheological properties of the net-like red soil.

In recent decades, significant efforts have been devoted to building up constitutive models to describe the time-dependent behaviors of clay^9–12^, but little focus on the stress relaxation process. The element rheology models (such as the generalized Kelvin model, Burgers model, and Nishihara model) with a capability to account for the elastic-viscoplastic behavior were verified by some researchers^13–15^. However, those conventional element models sometimes have difficulties precisely describing the nonlinear characteristics of soil stress relaxation because the parameters of the viscous element cannot change with time at all^16^. So the fractional derivative model was recently employed to construct a time-dependent constitutive model^17–22^, and some beneficial progress has been made. Nevertheless, these fractional constitutive models mainly focused on rock mass creep and stress relaxation behaviors, little on soil stress relaxation, let alone variable-order fractional derivative relaxation model that could reflect the whole stress relaxation process. Moreover, the creep model of fractional order for rock mass is unsuitable for the characterization of nonlinear relaxation behaviors of clay. To this end, Wang et al.^23^ presented fractional relaxation models of the Voigt and Maxwell model in series and the Voigt and Maxwell model in parallel based on the data obtained from experiments performed on the stage-loading mode.

In addition, soil damage gradually accumulates, and the property of the material is time-dependent under loading, so the order of fractional derivatives should be variable^24^. However, traditional fractional models are mainly proposed based on constant fractional calculus and disregard the damage to the soil. These models are not sufficient to fully simulate the different stages of relaxation. At the same time, the rheology of geotechnical materials is the process of the relative movement of particles inside the soil or rock mass and the gradual expansion and adjustment of micro cracks, so it is necessary to consider the damage time effect when studying the constitutive equation. Recently, Zhou et al.^25^ and Liu et al.^26^ proposed constitutive damage models of rock based on fractional calculus theory and damage variables. Although the research of fractional rheological models has made beneficial progress, the study is mostly for rocks. And the models are mostly fixed fractional models, coupled damage models are rarely discussed, and the research on soil is relatively minor. These models enable us to depict the whole relaxation progress of the clay to a certain extent. However, these conventional fractional derivative models with the fixed order cannot fully reflect the time-dependent property of clay under loading^27,28^. Up to the present, in the description of the relaxation behavior of soil, variable-order fractional relaxation models with clear physical meaning are relatively rare. The recently variable-order fractional theory has been adopted to analyze rock rheological properties^29–33^. These researchers found that the order of the fractional elements can represent different creep stage features of the rock. Therefore, exploring a variable-order-based constitutive model of soil relaxation will benefit the practical characterization of soil relaxation and provide a new idea for studying the soil relaxation model. The extension of fractional calculus with the variable order is addressed here to exhibit the evolution of relaxation properties of the net-like red soil.

Meanwhile, some long-term stress relaxation experiments were performed on the rock^15,16,19,34^, clay^35^, and sand^36,37^ through improved laboratory equipment. Considerable attention also had been directed to effects of the loading mode^35^, the loading rate^38–41^, the dynamic loading^42^, the water content^43^, the axial strain level^8^, and the confining pressure^44–46^ on the time-dependent behavior of saturated soils. Recently, Wang et al.^23^ investigated the relaxation behaviors of the unsaturated net-like red soil with triaxial experiments under the stage-loading mode. Still, investigations have not involved the step-loading way. However, the step-loading way of the pre-strain contributes to exploring the cumulative effects of the pre-strain on the relaxation behavior of the same specimen^40^. So, although some previous researches had been discussed for the characteristics of stress relaxation of soil, the stress relaxation under the step-loading mode has not yet reached the same understanding for the net-like red soil.

This study aims to understand better the stress relaxation behavior of the net-like red soil. Herein, a novel variable-order fractional derivative model for the consideration of damage evolution was proposed to depict the real progress of stress relaxation for the net-like red soil. Then, unsaturated triaxial stress relaxation tests under the step-loading mode were performed to confirm the validity of the proposed model and further investigate the nonlinear relaxation characteristics of the tested soil. Moreover, comparisons with other analytical models were further discussed to verify the correctness and rationality of the variable-order fractional relaxation model. These results will highlight the stress relaxation behavior of net-like red soil and provide a basis for analysis of the long-term stability of the earth.

## Methods

As we know, macroscopic nonlinear characteristics of relaxation in the net-like red soils are the embodiment of the damage effects of internal structure. In addition, it is challenging to describe nonlinear effects with a simple empirical relaxation model or an element model of integer order. The fractional derivative theory is a powerful way for building the time-dependent constitutive model^17,20,43^. It can make up for the insufficiency of the conventional element models that cannot accurately reflect the nonlinear characteristics of stress relaxation. Therefore, the variable-order fractional relaxation model is discussed here to describe better the nonlinear relaxation process for the net-like red soil.

Fractional calculus can be defined in different ways. The most extensive forms of fractional calculus are Riemann–Liouville, Caputo, and Grunwald–Letnikov fractional calculus. As we know, Riemann–Liouville (R-L) fractional derivative is first to integrate the function and then obtain the result. The Caputo fractional derivative is to direct the function first and then perform the integral^47,48^. Therefore, from a mathematical perspective, the Caputo fractional derivative has higher requirements for operations. The R-L fractional derivative has a particular super singularity. Still, the soil fractional relaxation model based on the R-L fractional derivative does not involve the singularity problem. The Caputo fractional derivative itself has weak singular nature. However, the soil fractional relaxation model based on the Caputo fractional derivative is challenging, so the R-L fractional derivative was adopted to construct the fractional relaxation model for the net-like red soil. To date, many definitions have been adopted to explain the fractional derivative theory. The Riemann–Liouville fractional calculus theory was used here to define fractional derivative,

1 $$\frac{{d^{\beta } f(t)}}{{dt^{\beta } }} = {}_{{t_{0} }}D_{t}^{\beta } f(t) = \frac{{d^{n} }}{{dt^{n} }}\int_{{t_{0} }}^{t} {\frac{{(t - \tau )^{n - \beta - 1} }}{\Gamma (n - \beta )}} f(\tau )d\tau .$$

where *t* is time; $t_{0}$ is initial time; $\beta$ is the order of fractional derivative, $\beta > 0$, and $n - 1 \le \beta < n$; *n* is the positive integer; ${}_{{t_{0} }}D^{\beta }_{t}$ is a fractional derivative of $\beta$ order; $\Gamma (n - \beta )$ is a Gamma function,

2 $$\Gamma (n - \beta ) = \int_{{0}}^{\infty } {e^{ - t} t^{n - \beta - 1} dt} .$$

The fractional calculus theory is recently used to depict the elementary models, including the Hookean, Newtonian, and composite models, especially for the rheology of viscoelastic and viscoplastic materials^34,49^. Abel dashpot is defined based on the Abel kernel. The fractional-order represents a typical application of fractional derivative description of viscoelastic materials by interpolating between the Newton dashpot and the spring^50^. The constitutive relationship of the Abel dashpot based on the R-L fractional derivative theory is given as,

3 $$\sigma (t) = \xi \frac{{d^{\beta } \varepsilon (t)}}{{dt^{\beta } }},$$

where *ξ* is the viscosity coefficient; *β* denotes the fractional-order. The Abel dashpot model of *β* = 0 can be regarded as a linear elastic solid, representing an ideal Newtonian fluid when the order *β* = 1^51^. It can be used to describe the characteristics of both solids and liquids and eliminates the shortcoming of an element being solely either a spring or the Newtonian dashpot^50^.

**Figure 1:**
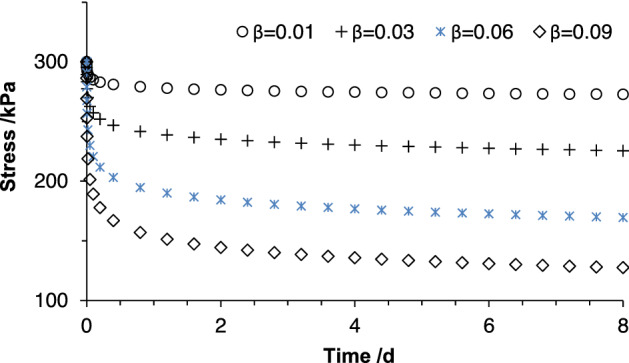
Time-dependent relaxation stress described by a fractional-order elementary model (Abe dashpot): *σ*_0_ = 300 kPa and *ε*_0_ = 5%.

As we know, the element with a constant strain behaves in the relaxation behavior. Let *n* = 1, *t*_0_ = 0, 0 < *β* ≤ 1, then according to the definition of fractional calculus, Eq. (3) for the stress relaxation can be written as

4 $$\sigma (t) = \xi \frac{{d^{\beta } \varepsilon (t)}}{{dt^{\beta } }}{ = }\frac{{\xi \varepsilon_{0} }}{\Gamma (1 - \beta )} \cdot \frac{d}{dt}\int_{0}^{t} {(t - \tau )^{ - \beta } d\tau } = \frac{{\xi \varepsilon_{0} t^{ - \beta } }}{\Gamma (1 - \beta )}$$

where *ε*_0_ is a constant axial strain; *t* is the period of stress relaxation. Equation (4) represents the relaxation stress characterized by the Abel dashpot. Substituting initial stress σ_0_ = 300 kPa at the axial strain ε_0_ = 5% into Eq. (4), one finds a series of stress relaxation curves as shown in Fig. 1. It is seen from Fig. 1 that the clay behaves like the dependence of relaxation stress under various fractional orders. This fractional-order elementary model with a larger fractional-order tends to exhibit the fluid properties, while one with a smaller fractional-order shows a complex property. This indicates that the Abel dashpot can respond over multiple time scales relative to the exponential time dependence in spring and dashpot models^52^.

When *n* ≥ 2, *t*_0_ = 0, 0 < *β* ≤ 1, from Eqs. (1) and (3), the corresponding constitutive model for the stress relaxation is given as,

5 $$\sigma (t) = \xi \frac{{d^{\beta } \varepsilon (t)}}{{dt^{\beta } }}{ = }\frac{{\xi \varepsilon_{0} }}{\Gamma (n - \beta )} \cdot \frac{{d^{n} }}{{dt^{n} }}\int_{0}^{t} {(t - \tau )^{n - \beta - 1} d\tau } = \frac{{\xi \varepsilon_{0} t^{ - \beta } }}{\Gamma (n - \beta )}\prod\limits_{k = 1}^{n - 1} {(n - k - \beta )} ,$$

where *k* is a positive integer, *k* = 1, 2, …, *n*.

## Formulation of analytical model

### Relaxation model based on variable-order fractional derivatives theory

As discussed above, the relaxation model of fixed fractional-order can overcome shortcomings of the integral-order relaxation model. Still, it cannot exhibit the evolution of time-dependent soil characteristics. So the fractional model of variable-order $\alpha$ changing with different relaxation stages is addressed here to depict nonlinear features of stress relaxation in soil. Similar to the constant-order fractional derivative, the generalized R-L fractional derivative operator of variable-order in the specific time interval is expressed as,

6 $${}_{{t_{0} }}D^{\alpha (t)}_{t} = \frac{{d^{n} }}{{dt^{n} }}\int_{{t_{k - 1} }}^{{t_{k} }} {\frac{{(t - \tau )^{n - \alpha (t) - 1} }}{{\Gamma \left[ {n - \alpha (t)} \right]}}f(\tau )d\tau } .$$

where *α*(*t*) denote the fractional-order in the *k*th time interval [*t*_*k−*1_, *t*) as listed in Table 1, the variable-order fractional elementary model can describe the characteristic of time memory that the results of past calculations have significant influences on the current results.

**Table 1:** Values of fractional order and viscosity coefficient versus time.

Time	Fractional order *α*(*t*)	Viscosity coefficient
0 ≤ *t* < *t*_1_	*α*_1_	*ξ*_1_
*t*_1_ ≤ *t* < *t*_2_	*α*_2_	*ξ*_2_
$\cdots$	$\cdots$	$\cdots$
*t*_*k−*1_ ≤ *t* < *t*_*k*_	*α*_*k*_	*ξ*_*k*_
$\cdots$	$\cdots$	$\cdots$
*t*_*n−*1_ ≤ *t* ≤ *t*_*n*_	*α*_*n*_	*ξ*_*n*_

In practice, the mechanical properties of the soil change with time during the relaxation process. Theoretically, variable-order fractional calculus is a direct extension of fractional calculus, and the order should change with time. For practical application, the fractional order within each relaxation stage is assumed as a constant to simplify the fractional computation procedure. Therefore, considering that *n* = 1, *t*_0_ = 0, 0 < *β* ≤ 1, *ε*(*t*) = constant = *ε*_0_, the fractional stress relaxation model in the specific time interval is,

7 $$\sigma (t) = \frac{{\xi \varepsilon_{0} t^{ - \alpha (t)} }}{{\Gamma \left[ {1 - \alpha (t)} \right]}}.$$

where *σ* is the stress, kPa; *ξ* denotes the viscosity coefficient, kPa·d; *ε*_0_ is the pre-strain; *t* is the relaxation time, d; *ɑ* is the order of fraction, *ɑ* ∈ [0, 1]. For the Kelvin model, let the applied stress of the Abel dashpot (soft-matter element) be *σ*_2_, then the constitutive relation of the Abel dashpot is

8 $$\sigma_{2} (t) = \sum\limits_{k = 1}^{n} {\varepsilon_{0} } \frac{{\xi_{k} (t_{k} - t_{k - 1} )^{{ - \alpha_{k} }} }}{{\Gamma (1 - \alpha_{k} )}},\;0 \le \alpha_{k} \le 1,\;t_{k - 1} \le t < t_{k} .$$

Assuming that the applied stress and elastic modulus of spring in the Kelvin model are *σ*_1_ and *E*_0,_ respectively, then the Kelvin fractional relaxation model of variable order under the uniaxial loading is obtained as

9 $$\sigma = \sigma_{1} + \sigma_{2} = E_{0} \varepsilon_{0} + \sum\limits_{k = 1}^{n} {\varepsilon_{0} } \frac{{\xi_{k} (t_{k} - t_{k - 1} )^{{ - \alpha_{k} }} }}{{\Gamma (1 - \alpha_{k} )}}.$$

### Nonlinear damage model of stress relaxation

Measured stress relaxation curves at various strains applied to the specimen before the relaxation process present a similar relaxation pattern^40^. Luckily, this enables us to depict the stress relaxation behavior of the net-like red soil by an analytical model. Some studies have developed elementary models of variable parameter methods to investigate the effect of initial damage on the relaxation behavior of rock mass^53^ and got some benefits. Still, up to date, few studies have paid attention to the nonlinear rheological problems of soil. On the other hand, composite elementary models with many parameters are not easy to apply to real problems because modeling with more parameter numbers is challenging to determine model parameters under complex stress conditions^54,55^ and may give rise to calculation difficulty. From this consideration, some fractional elementary models have been presented to improve these analytical models; however, those models almost do not consider the effect of fractional order and time-dependent parameters^21^ and sometimes ignore the macro characteristics determined from test data. Material elasticity and viscoelasticity features vary according to the tests^56^. A good analytical model should be suitable for describing different kinds of viscoelastic properties^57^. Thus, a nonlinear analytical model with variable parameters is more accurate to reflect the rheological properties than a steady model with constant parameters^58^. To further investigate the nonlinear relaxation behavior of net-like red soil, a fractional damage model with variable orders is addressed to reflect the inherent relaxation mechanisms considering the damage and fracture properties.

Numerous studies^16,59–62^ have shown that the evolution of rheological damage can be assumed as a negative exponential function when considering only the effect of the time of load action. Therefore, a negative exponential form of the damage variable is also used here to characterize the relaxation damage. i. e.

10 $$D = 1 - e^{ - \varpi t} .$$

where *D* denotes the damage variable; *ω* represents a constant about the material property. The results of the relaxation test of net-like red soil^40^ indicate that the relaxation process shows negative exponential decay. Liu et al. found that for the mica-quartz schist, its damage equation obeys the negative exponential form considering energy dissipation^62^. Therefore, the damage variable *D* in Eq. (10) also reflects the energy dissipation uniformly. Recently, Okuka and Zorica^63^ examined the thermodynamically consistent fractional models. Moreover, the previous studies indicate that the viscosity coefficient versus time obeys the attenuation law of the negative exponent^64^. Consequently, the damage variable is incorporated here into the fractional element to reflect the damaging effect on stress relaxation as follows,

11 $$\xi (D,\;t) = \xi_{0} (1 - D).$$

where *ξ*_0_ denotes the initial viscosity coefficient during the relaxation process. Thus, from Eqs. (9) to (11), the relaxation damage model of variable fractional-order is given as,

12 $$\sigma = E_{0} \varepsilon_{0} + \sum\limits_{k = 1}^{n} {\varepsilon_{0} } \frac{{\xi_{k} e^{{ - \omega_{k} t}} (t_{k} - t_{k - 1} )^{{ - \alpha_{k} }} }}{{\Gamma (1 - \alpha_{k} )}}.$$

Generally, net-like red soil's measured relaxation curves consist of two relaxation stages^23^ with s segment point. So, through Eq. (12), the constitutive relaxation model corresponding for the initial stage under the uniaxial loading is,

13 $$\sigma = E_{0} \varepsilon_{0} + \varepsilon_{0} \frac{{\xi_{1} e^{{ - \omega_{1} t}} (t - t_{0} )^{{ - \alpha_{1} }} }}{{\Gamma (1 - \alpha_{1} )}}, \, 0 = t_{0} \le t \le t_{1} ,$$

and for the second stage,

14 $$\sigma = E_{0} \varepsilon_{0} + \varepsilon_{0} \frac{{\xi_{1} e^{{ - \omega_{1} t_{{1}} }} \left( {t_{1} { - }t_{{0}} } \right)^{{ - \alpha_{1} }} }}{{\Gamma (1 - \alpha_{1} )}} + \varepsilon_{0} \frac{{\xi_{2} e^{{ - \omega_{2} t}} (t - t_{{1}} )^{{ - \alpha_{2} }} }}{{\Gamma (1 - \alpha_{2} )}},\;t_{1} \le t < t_{2} .$$

### Three-dimensional constitutive model of stress relaxation

The soil is usually in a three-dimensional stress state, so establishing a three-dimensional stress relaxation constitutive model is helpful to analyze the long-term stability of net-like red soil. As we know, the internal stress tensor *σ*_*ij*_ can be decomposed into a spherical stress tensor *σ*_*m*_ and deviatoric stress tensor *S*_*ij*_; the corresponding total strain tensor *ε*_*ij*_ consists of the spherical strain tensor *ε*_*m*_ and deviatoric strain tensor *e*_*ij*_, and their constitutive relations are

15 $$\sigma_{ij} = S_{ij} + \delta_{ij} \sigma_{m} = S_{ij} + \frac{1}{3}\delta_{ij} \sigma_{kk} ,$$

16 $$\varepsilon_{ij} = e_{ij} + \delta_{ij} \varepsilon_{m} { = }e_{ij} + \frac{1}{3}\delta_{ij} \varepsilon_{kk} ,$$

where *δ*_*ij*_ is the Kronecker delta. If the spherical stress contributes to the volume changes and the deviatoric strain mainly causes the stress relaxation, then from Eq. (13), three-dimensional constitutive equations of Kelvin viscoelastic body for the initial relaxation stage can be given as,

17 $$\sigma_{m} = 3K\varepsilon_{m}$$

18 $$S_{ij} = 2e{}_{ij}(G + \frac{{\xi_{1} e^{{ - \omega_{1} t}} (t_{1} - t_{0} )^{{ - \alpha_{1} }} }}{{\Gamma (1 - \alpha_{1} )}}),\;0 = t_{0} \le t \le t_{1} .$$

where *K* and *G* denote the bulk modulus and shear modulus, respectively. Similarly, from Eq. (14) the deviatoric stress for the second relaxation stage is,

19 $$S_{ij} = 2e_{ij} \left\{ {G + \frac{{\xi_{1} e^{{ - \omega_{1} t}} t_{1}^{{ - \alpha_{1} }} }}{{\Gamma (1 - \alpha_{1} )}} + \frac{{\xi_{2} e^{{ - \omega_{2} t}} (t - t_{2} )^{{ - \alpha_{2} }} }}{{\Gamma (1 - \alpha_{2} )}}} \right\},\;t_{1} \le t < t_{2}$$

## Parameter determination and verification of proposed models

### Tested soils and test equipment

**Figure 2:**
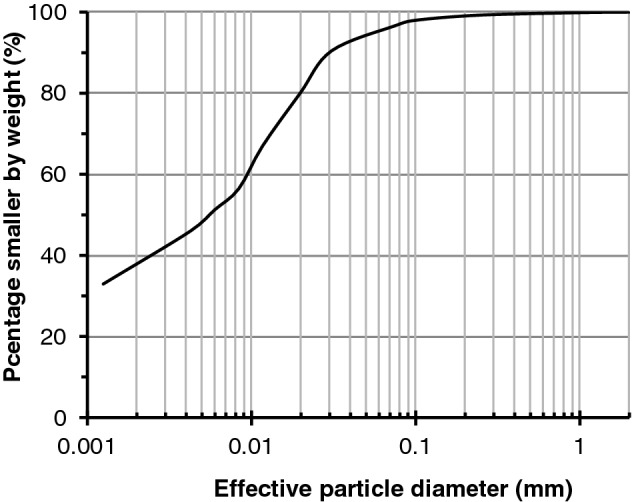
Particle size distribution curve.

**Figure 3:**
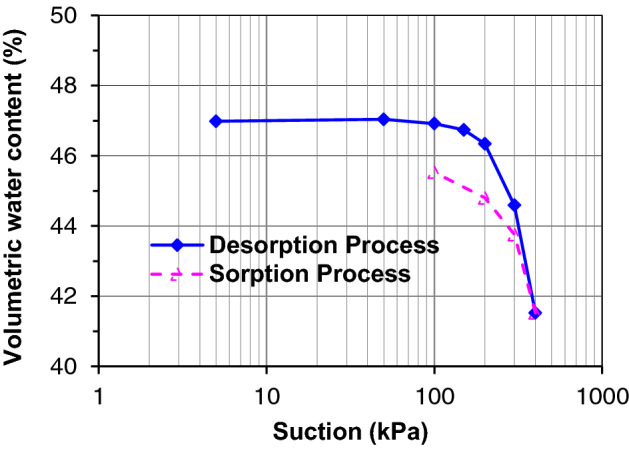
Soil–water characteristics curve.

Triaxial stress relaxation experiments were performed on the net-like red soil to verify the validity of the proposed model. The physical properties of the tested soil were listed in Table 2. The particle size distribution was illustrated in Fig. 2. The main components of clay minerals were illite and kaolinite by X-ray diffraction (XRD) experiments^2^. The soil–water characteristics curve (SWCC), as shown in Fig. 3, was also determined using the GDS triaxial stress path testing system developed by Ng et al.^65^ using axis-translation technology. Remolded cylindrical remolded specimens with a diameter of 38 mm and a length of 80 mm were prepared by the following steps. The net-like red soil is first ground to powder and dried in an oven before the specimen preparation. Secondly, add the de-aired water to attain the given moisture content of 18% and dry density of 1810 kN/m^3^, and keep the soil in closed plastic boxes over 24 h for complete saturation. Finally, cylindrical specimens are prepared with special compaction equipment.

**Table 2:** Physical properties of tested net-like red soil.

Maximum dry density (g·cm^−3^)	Liquid limit (%)	Plasticity limit (%)	Plasticity index	Optimum water content (%)	Moisture content (%)
1.81	48.5	28.2	20.0	17.9	23.6

All triaxial stress relaxation tests with a step-loading mode were performed by a digital geotechnical system (GDS) with the high accuracy of the 1.0 kPa pressure resolution and the 1.0 mm^3^ volume resolution. The given strain level was first applied rapidly on the specimen with a steady strain rate and maintained constant, then the magnitude of axial stress was determined with time until reaching a relatively stable state of the relaxation process, and the next strain level was applied on the specimen to continue the follow-up relaxation test. The unsaturated relaxation experiment's testing procedure consists of four steps: sample installation, consolidation and suction equilibrium, pre-strain control, and relaxation. Detailed methods can be found in the reference^40^. Suctions of 50 kPa, 100 kPa, and 150 kPa were applied on specimens to examine responding effects on the stress relaxation behaviors of the net-like red soil, and the testing parameters were listed in Table 3.

**Table 3:** Parameters of relaxation tests.

Case	Axial strains (%)	Confining pressure (kPa)	Pore air pressure (kPa)	Back pressure (kPa)	Suction (kPa)
A	0.5 → 1.0 → 1.5 → 2.0	305	250	200	50
B	0.5 → 1.0 → 1.5 → 2.0	305	250	150	100
C	0.5 → 1.0 → 1.5 → 2.0	305	200	50	150

### Results of stress relaxation experiments

**Figure 4:**
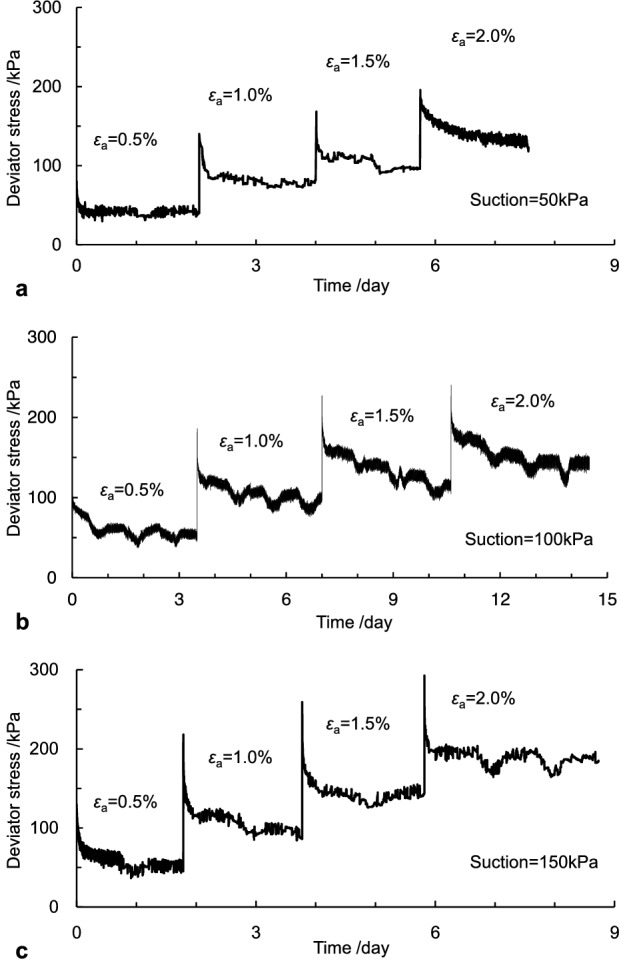
The measured stress relaxation curves: (**a)** suction = 50 kPa, (**b)** suction = 100 kPa and (**c)** suction = 150 kPa.

**Figure 5:**
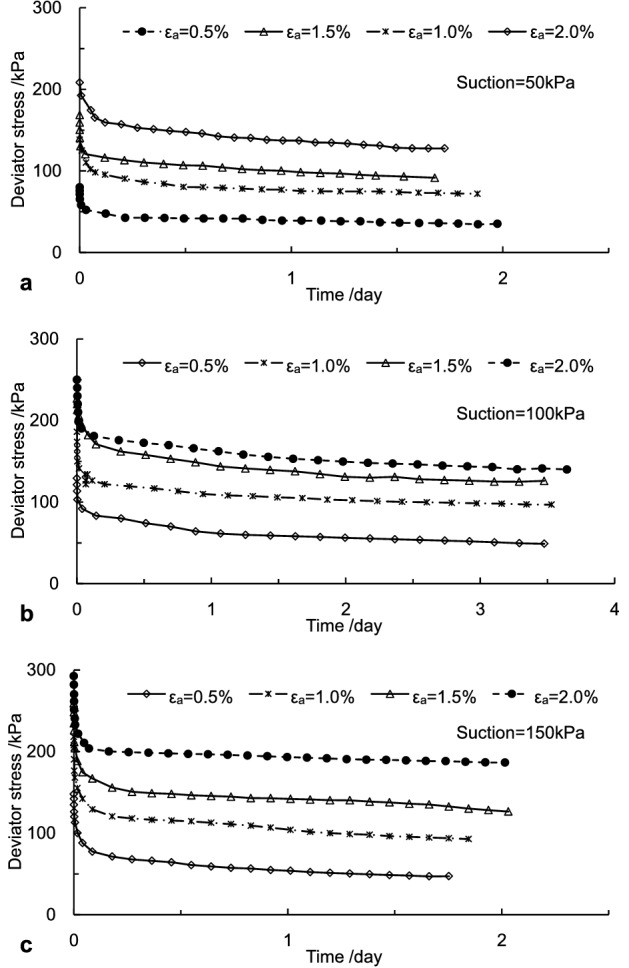
The processed stress relaxation curves: (**a)** suction = 50 kPa, (**b)** suction = 100 kPa and (**c)** suction = 150 kPa.

The measured stress relaxation curves with various suction and the same confining pressure are illustrated in Fig. 4. It was observed in Fig. 4 that the measured curves exhibited oscillatory features with time, which might be caused by the temperature difference between day and night. In addition, the obtained stress relaxation data were enormous because the sampling interval of data was 10 s, and the duration of experiments was over seven days. The smoothing process was performed to reduce data oscillations. The corresponding processed curves at various pre-strain levels under suctions of 50 kPa, 100 kPa, and 150 kPa were presented in Fig. 5.

**Figure 6:**
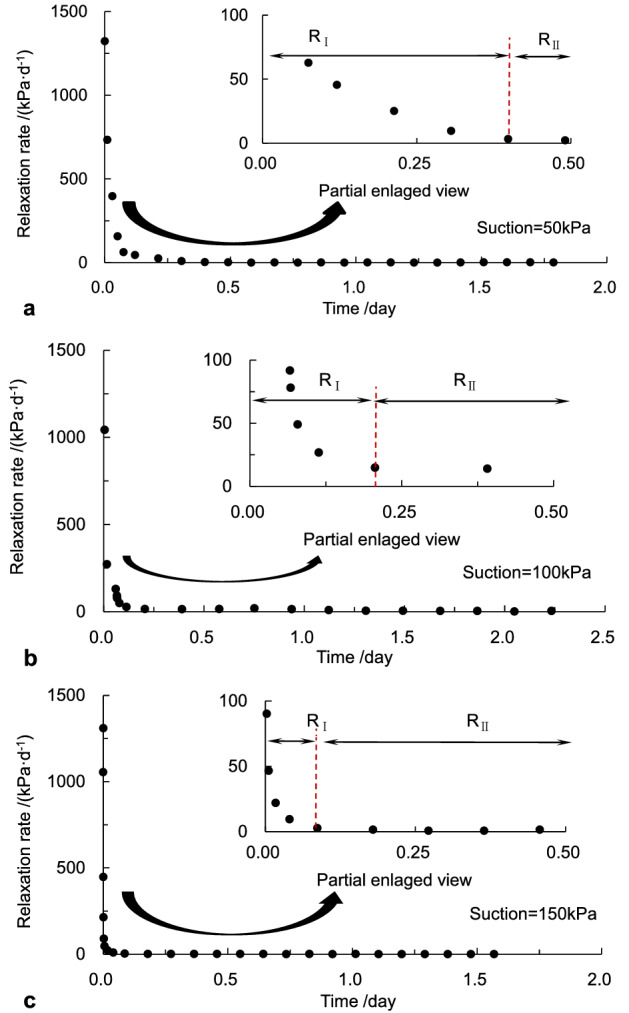
The characteristics of relaxation rate at the 1% strain: (**a)** suction = 50 kPa, (**b)** suction = 100 kPa and (**c)** suction = 150 kPa.

As shown in Fig. 5, the relaxation curves showed a similar pattern despite various suctions. The stress relaxation process was mainly divided into two stages: rapid relaxation stage (*R*_Ι_) and attenuated relaxation stage (*R*_II_) according to the relaxation rate. Namely, the initial phase of stress relaxation was the fast relaxation process that the deviator stress reduced at a sharply decreasing rate with the constant axial deformation; the relaxation underwent the second stage when the deviator stress fell at a steady speed. The relaxation rate curves and locally magnified curves versus time at the 1.0% strain were illustrated in Fig. 6. It was seen that in the rapid relaxation stage, the deviator stress dropped rapidly, and the relaxation rate was much more prominent. The relaxation process was relatively fast in the rapid relaxation stage, and the amount of relaxation stress was considerable. While in the attenuated relaxation stage, the relaxation rate was slow, and the axial stress in the specimen continued to decrease gradually with time until the steady-state was reached. It was also seen in Fig. 6 that the whole relaxation times for cases A, B, and C were about 1.88 days, 3.53 days, and 1.85 days, respectively. Therefore, the pattern of relaxation stages for the specimens applied on the same axial strain level was similar despite various suctions. Still, there were great influences on the relaxation rate and the duration time of the initial relaxation stage. The duration of the rapid relaxation stage decreased with the increment of the suction. Still, it behaved like nonlinear characteristics because soil structure and damage evolution are complex in the unsaturated soil.

The relaxation magnitude and duration time in two relaxation stages under different suctions were different, as listed in Table 4. The time at the segment point of two relaxation stages was 9.56 h, 4.94 h, and 2.08 h, respectively, for the relaxation experiments subjected to suctions of 50 kPa, 100 kPa, and 150 kPa. The amount of stress relaxation at the time of 0.398 days in the first stage of case A is approximately 81.1% of the total relaxation magnitude, while around 73.8% at 0.206 days in B. About 71.5% at the time of 0.087 days in case C. Concepts of the relaxation magnitude and residual stress ratio were introduced here to interpret the impact of the suction on the stress relaxation behavior of the net-like red soil. Responding definitions of the relaxation magnitude and residual stress ratio are,

20 $$\Delta \sigma = \sigma_{0} - \sigma_{t} ,$$

21 $$\zeta { = }{{\sigma_{t} } \mathord{\left/ {\vphantom {{\sigma_{t} } {\sigma_{0} }}} \right. \kern-\nulldelimiterspace} {\sigma_{0} }}.$$

where *σ*_0_ is the initial axial deviator stress; *σ*_*t*_ denotes the residual deviator stress in the specimen; Δ*σ* denotes the variation magnitude of the deviator stress in the sample due to the stress relaxation; *ζ* represents the ratio of the residual stress. The residual stress ratios in the specimens at different pre-strain levels under suctions of 50 kPa, 100 kPa and 150 kPa, were listed in Table 5.

**Table 4:** The characteristics in two relaxation stages.

Case	The rapid relaxation stage			The attenuated relaxation stage		
	The initial deviator stress (kPa)	Relaxation magnitude (kPa)	Duration (day)	The initial deviator stress (kPa)	Relaxation magnitude (kPa)	Duration (day)
A	138.23	53.91	0.398	84.32	12.31	1.482
B	186.08	63.11	0.206	122.97	22.38	3.324
C	218.21	88.95	0.087	129.26	35.53	1.759

**Table 5:** Characterization parameters of stress relaxation.

Suction (kPa)	Pre-strain (%)	Initial deviator stress (kPa)	Residual deviator stress (kPa)	Relaxation magnitude (kPa)	Residual stress ratio
50	0.5	80.50	35.21	45.29	0.44
	1.0	138.23	72.01	66.22	0.52
	1.5	168.43	97.44	70.99	0.58
	2.0	208.32	127.62	80.7	0.61
100	0.5	129.15	48.51	80.64	0.38
	1.0	186.08	96.85	89.23	0.52
	1.5	227.10	126.04	101.06	0.55
	2.0	250.02	140.30	109.72	0.56
150	0.5	147.92	41.73	106.19	0.28
	1.0	218.21	95.50	122.71	0.43
	1.5	250.26	125.12	125.14	0.50
	2.0	298.32	169.25	129.07	0.57

It was found that the specimen of a higher pre-strain exhibited the more significant amounts of the residual stress ratio. More energy was released from the increasing relaxation stress, while the residual stress ratio decreased despite the increment of the relaxation stress. In addition, the initial value of deviator stress and the relaxation magnitude both increased with the suction under the same strain level. These results suggest that suction plays a considerable role in the stress relaxation behavior of unsaturated net-like red soil.

### Parameters identification of model and validation

**Figure 7:**
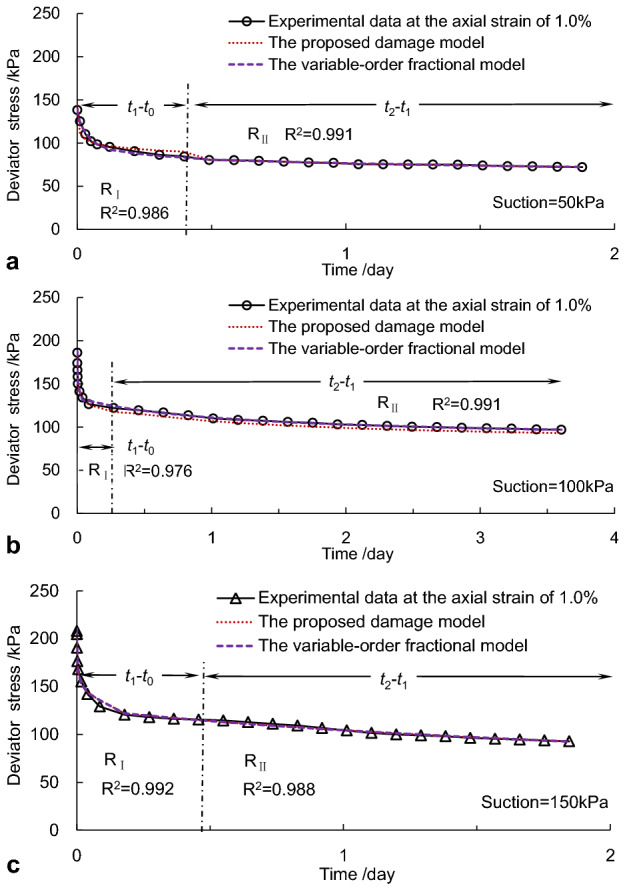
Comparison of simulated curves with measured curves: (**a)** case A, (**b)** case B, and (**c)** case C.

The measured relaxation curves, which were obtained from the specimens at the step loading strain of 1.0%, were taken to verify the effectiveness and correctness of the proposed model. The Levenberg–Marquardt (L-M) method was used to identify the parameters of analytical models, as listed in Table 6. The square of the correlation coefficient, *R*^2^, was also undertaken to discuss the validation of the model. Fitting curves for the damage model of variable fractional-order were depicted as shown in Fig. 7.

**Table 6:** Fitted parameter values for the proposed damage model.

Suction (kPa)	Strain (%)	*E*_0_ (kPa)	*ξ*_1_ (kPa·day)	*ω*_1_	*α*_1_	*ξ*_2_ (kPa·day)	*ω*_2_	*α*_2_	*R*_Ι_^2^	*R*_II_^2^
50	0.5	39.21	9.64	2.70	0.17	108.27	4.43	0.22	0.992	0.997
	1.0	42.31	25.32	4.21	0.21	241.32	6.34	0.25	0.986	0.991
	1.5	65.32	30.21	5.32	0.24	261.01	8.96	0.32	0.997	0.980
	2.0	78.31	28.31	8.54	0.16	201.26	9.87	0.21	0.985	0.997
100	0.5	22.59	59.34	0.33	0.07	78.12	0.12	0.11	0.995	0.997
	1.0	33.31	27.47	0.14	0.10	301.74	0.48	0.20	0.976	0.991
	1.5	51.02	15.85	6.73	0.05	28.83	0.75	0.17	0.997	0.980
	2.0	62.31	43.83	0.04	0.05	50.01	0.02	0.67	0.965	0.992
150	0.5	113.25	81.32	45.32	0.09	105.32	0.46	0.11	0.993	0.994
	1.0	135.23	85.21	34.21	0.12	112.31	0.54	0.16	0.990	0.986
	1.5	156.21	78.45	89.32	0.21	214.32	0.25	0.23	0.997	0.997
	2.0	189.32	94.12	268.32	0.13	102.31	0.69	0.16	0.965	0.991

It was seen in Fig. 7 that the fitting curves of the proposed damage model did good agreements with the measured curves. And the fractional derivative damage model of variable-order was more accurate than the variable-order fractional derivative model for depicting the nonlinear characteristics of stress relaxation, especially in describing the initial relaxation stage. Moreover, fitting correlation coefficients of relaxation stages under different suction levels were all over 0.98.

It was seen from Table 6 that the instantaneous elasticity modulus *E*_0_ increased with the increment of strain level and suction. The variation of the instantaneous elasticity modulus at different pre-strain during the relaxation process reflected the changes in strength. The instantaneous elasticity moduli of specimens with 150 kPa suction were generally greater than samples with 100kPa or 50 kPa suction under the same pre-strain. These results confirmed the results obtained from Table 5. The accumulated energy of the specimen with 150 kPa suction and 105 kPa net confining pressure is much greater than those with 50 kPa or 100 kPa suction and the confining pressure of 50 kPa. The reason may be because the structure of the sample with a high suction may be more challenging to rearrange in the rapid relaxation stage. The values of the viscosity coefficient *ξ* and the fractional-order *α* were correspondingly much smaller in the first relaxation stage than those in the subsequent relaxation stage. In the attenuation relaxation stage, the time effects of the net-like red soil behaved more viscosity on account of the new micro-cracks and fractures that occurred in the specimen. Furthermore, fitting correlation coefficients of two relaxation stages at different strain levels ranged from 0.965 to 0.997. This indicates that the Kelvin relaxation damage model of variable fractional-order proposed here can depict the nonlinear characteristics of stress relaxation in the soil.

## Comparisons

**Figure 8:**
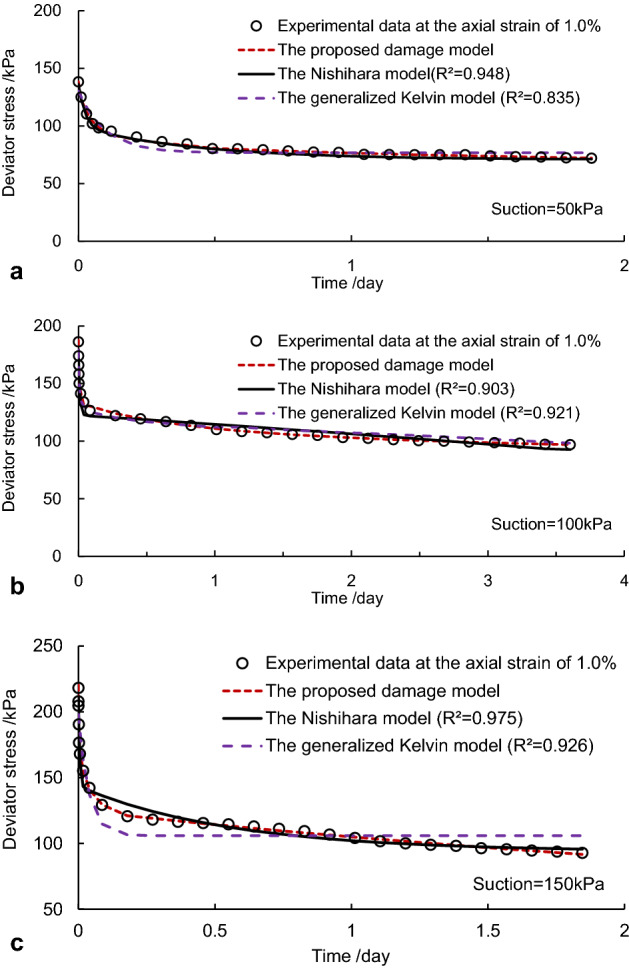
Comparisons of fitting curves by the proposed model and other models: (**a)** case A, (**b)** case B and (**c)** case C.

The validity of the proposed model was further verified by comparisons of fitting curves from the Nishihara model and the generalized Kelvin model. The fit curves obtained from the above models were depicted as shown in Fig. 8. Fitting correlation coefficients of two relaxation stages were all over 0.97 in the proposed model and more significant than the Nishihara model. The generalized Kelvin model was presented, as shown in Fig. 8. It was also seen in Fig. 8 that the fitting curves deviated considerably from the experimental data that existed in case of B for the Nishihara model and the generalized Kelvin model, and apparent deviations as well occurred in cases A and C, especially for the generalized Kelvin model. This indicates that the conventional Nishihara and generalized Kelvin models may have difficulty precisely depicting nonlinear characteristics and the whole relaxation process in the net-like red soil because the relationship of parameters of the viscous elements in those models cannot change with time at all.

## Sensitivity analysis of fractional order

**Figure 9:**
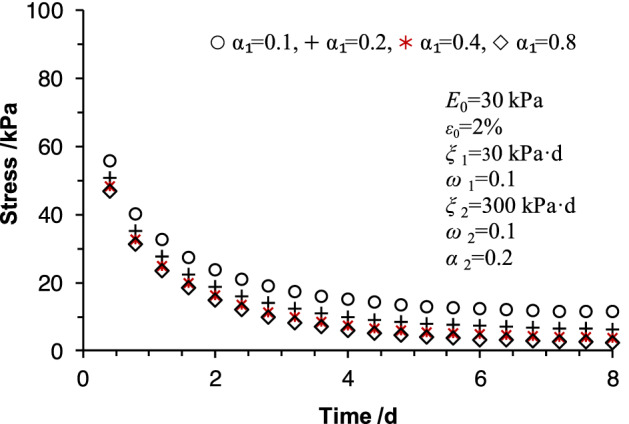
Sensitivity analysis of fractional order *α*_1_.

**Figure 10:**
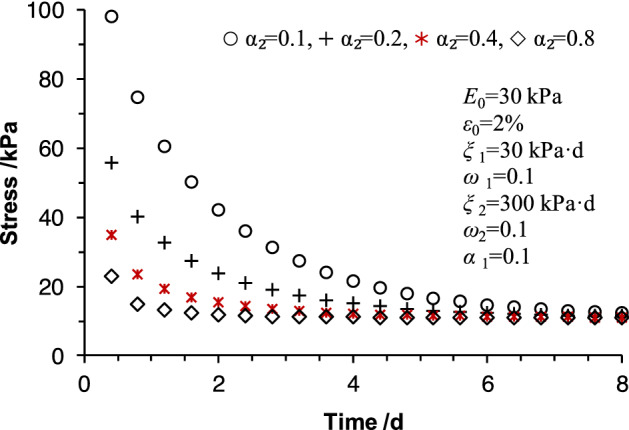
Sensitivity analysis of fractional-order *α*_2_.

To analyze the influence of the fraction orders on the present model, the various orders with same other parameters were adopted to conduct the sensitivity analysis. The corresponding results are illustrated in Figs. 9 and 10.

Figure 9 presents the results of the sensitivity analysis of fractional-order *α*_1_. The results were obtained with the same parameters of *E*_0_ = 30 kPa, *ε*_0_ = 2%, *ξ*_1_ = 30 kPa·d, *ω*_1_ = 0.1, *ξ*_2_ = 300 kPa·d, *ω*_2_ = 0.1, and *α*_2_ = 0.2. As shown in Fig. 9, the stress increased with the increment of the fraction order. Stress difference values between models of different fractional orders vary little over time. Figure 10 shows the results of the sensitivity analysis of fractional-order *α*_2_. The results were obtained with the same parameters of *E*_0_ = 30 kPa, *ε*_0_ = 2%, *ξ*_1_ = 30 kPa·d, *ω*_1_ = 0.1, *ξ*_2_ = 300 kPa·d, *ω*_2_ = 0.1, and *α*_1_ = 0.1. As shown in Fig. 10, the stress difference among models of different fractional orders is significant in the initial segment and decreases over time but decreases over time. The overall effect is more potent than the fractional-order *α*_1_. The results indicate that a higher value of the derivative order usually results in minor stress. Fractional order affects the magnitude of the relaxation stress, and the fractional-order *α*_2_ is relatively large.

## Conclusions

Stress relaxation is a primary rheological behavior of soil. However, more efforts have been made for the creep characteristics of saturated soil, less for the stress relaxation behavior of the net-like red soil. Herein, the nonlinear stress relaxation model using the fractional derivative theory was discussed to simulate the nonlinear stress relaxation characteristics of the net-like red soil. Moreover, triaxial relaxation experiments of the unsaturated net-like red soil under a step-loading mode were performed to identify proposed models' parameters and investigate the relaxation stage's characteristics, the relaxation magnitude, and the residual stress ratio. Furthermore, comparisons with other models are carried out to verify the validity of proposed models. Some conclusions were drawn as follows.

- (1) The comparative analysis results show that the theoretical curves simulated by the variable-order fractional relaxation model are consistent well with the experimental data. Compared to the conventional relaxation model, the simulation results derived from the variable-order fractional model agree highly with the experimental data and exhibit the evolution of the relaxation properties of soil. The model proposed here shows the superior capacity to represent the nonlinear characteristics caused by damage evolution in the relaxation process of net-like red soil. It is simple in structure and provides a reliable simulation of altered relaxation stages of the experimental results through the segmentation treatment. These results indicate that the proposed model based on variable-order fractional derivatives is reasonable and reliable and provide a new way to establish the effective connection of relaxation strain level and differential order. In addition, it can thoroughly reveal the evolution rules by the changing fraction order during the whole relaxation process of net-like red soil, and overcomes the defects that the fixed fractional relaxation model does not consider the change of soil damage over time.
- (2) Comparisons of the fitting results show that the Kelvin damage relaxation model of variable fractional-order can better agree with the measured results than the Nishihara and the generalized Kelvin models without considering the damage. The proposed model can exhibit the characteristics of both spring and the Newtonian dashpot and effectively describe the nonlinear characteristics of stress relaxation for the net-like red soil. The case study also indicates that the proposed analytical model with the changing fractional order can effectively depict the evolution properties of the whole relaxation process.
- (3) The sensitivity analysis shows that the fractional-order affects the soil relaxation stress and affects different fractions. Overall, the fractional order is large and has a relatively small effect on relaxation stress.
- (4) The measured relaxation curves are mainly made up of two stages. The rapid and attenuation relaxation stages behaved like similar patterns in tested specimens. The initial deviator stress and relaxation stress values increased with the increment of suction, and the relaxation duration decreased with the increasing suction, but these characteristics behaved nonlinearly. This may be because more energy is released in the specimens with a higher strain, and the sample with greater suction may have more difficulties arranging and compacting the micro-pores and particles. The suction and the cumulative strain significantly influence the relaxation behaviors of the unsaturated net-like red soil. So time effects of the unsaturated soil should be considered comprehensively in soil engineering applications.

This study will provide some supports for better understanding the stress relaxation behavior of unsaturated net-like red soil. Although the measured curves of the stress relaxation appear similar patterns despite the suction variations in the unsaturated net-like red soil, incorporating suction and net confining pressure effects into the proposed analytical model still needs to be further clarified. In addition, the segmented fractional relaxation model with a determined value cannot elaborate the mechanism. To better explain the relaxation mechanism of soil relaxation, exploring the time-based variable fractional model remains to be further investigated in the future.
